# Artificial intelligence for ovarian cancer diagnosis via ultrasound: a systematic review and quantitative assessment of model performance

**DOI:** 10.3389/frai.2025.1649746

**Published:** 2025-11-05

**Authors:** Igor Garcia-Atutxa, Francisca Villanueva-Flores, Ekaitz Dudagotia Barrio, Javier I. Sanchez-Villamil, José Martínez-Más, Andrés Bueno-Crespo

**Affiliations:** ^1^Escuela Politécnica Superior, Universidad Católica de Murcia (UCAM), Murcia, Spain; ^2^Centro de Investigación en Ciencia Aplicada y Tecnología Avanzada, (CICATA) Unidad Morelos del Instituto Politécnica Nacional (IPN), Xochitepec, Mexico; ^3^University of Murcia, Murcia, Spain; ^4^Facultad de Medicina, Universidad Católica de Murcia (UCAM), Murcia, Spain

**Keywords:** systematic review, meta-analysis, artificial intelligence, ultrasound, ovarian cancer, early detection, deep learning

## Abstract

**Background:**

Early and accurate detection of ovarian cancer (OC) remains clinically challenging, prompting exploration of artificial intelligence (AI)-based ultrasound diagnostics. This systematic review and meta-analysis critically evaluate diagnostic accuracy, methodological rigor, and clinical applicability of AI models for ovarian mass classification using B-mode ultrasound.

**Methods:**

A systematic literature search following PRISMA guidelines was conducted in PubMed, IEEE Xplore, and Scopus up to December 2024. Eligible studies included AI-based ovarian mass classification using B-mode ultrasound, reporting accuracy, sensitivity, specificity, and/or area under the ROC curve (AUC). Data extraction, quality assessment (PROBAST), and meta-analysis (random effects) were independently performed by two reviewers. Heterogeneity sources were explored.

**Results:**

From 823 identified records, 44 studies met inclusion criteria, covering over 650,000 images. Pooled performance metrics indicated high accuracy (92.3%), sensitivity (91.6%), specificity (90.1%), and AUC (0.93). Automated segmentation significantly outperformed manual segmentation in accuracy and sensitivity, demonstrating standardization benefits and reduced observer variability. Dataset size minimally correlated with performance, highlighting methodological rigor as a primary determinant. No specific AI architecture consistently outperformed others. Substantial methodological heterogeneity and frequent risk-of-bias issues (limited validation, small datasets) currently limit clinical translation.

**Conclusion:**

AI models show promising diagnostic performance for OC ultrasound imaging. However, addressing methodological challenges, including rigorous validation, standardized reporting (TRIPOD-AI, STARD-AI), and prospective multicenter studies, is essential for clinical integration. This review provides clear recommendations to enhance clinical translation of AI-based ultrasound diagnostics.

## Graphical abstract

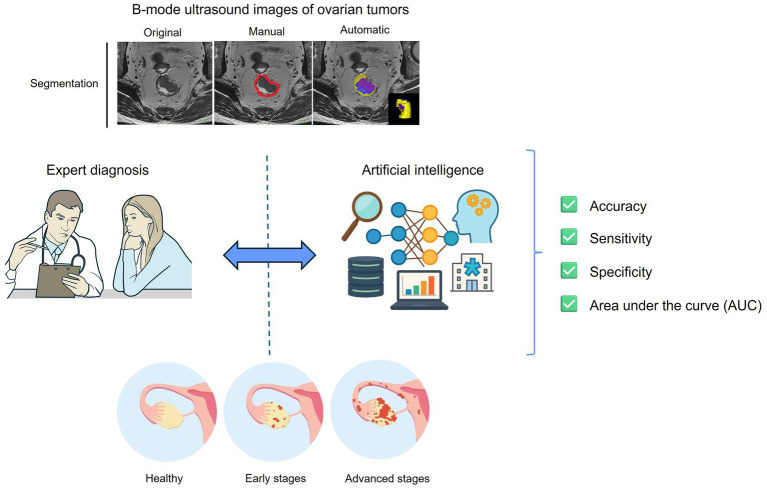

## 1 Introduction

Ovarian cancer (OC) is the most lethal gynecological neoplasm and the fifth leading cause of cancer-related mortality in women (Dalmartello et al., 2022; Siegel et al., 2021). The incidence of OC in the USA is estimated to be 10.2 cases, and the mortality rate is 6.0 deaths per 100,000 women per year, indicating approximately 1.1% lifetime risk for women (NIH, 2020). Despite advances in diagnosis and treatment, the mortality rate has not shown a significant decline over the past three decades, primarily due to the challenges in early detection and limited therapeutic efficacy in advanced-stage disease (Badgwell and Bast, 2007; Jacobs and Menon, 2004; Bast et al., 2007; Torre et al., 2018). Currently, 70% of OC cases are diagnosed at advanced stages, where five-year survival drastically drops to 20%–30%, in contrast to 80%–95% survival when detected at early stages (Bowtell et al., 2015). Therefore, improving early-stage detection methods is critically important to enhance patient outcomes.

Transvaginal ultrasound (TVS), computed tomography (CT), and magnetic resonance imaging (MRI) are the primary imaging modalities for OC detection. TVS is particularly advantageous as a non-invasive, cost-effective, accessible, and real-time imaging method, allowing assessment of ovarian masses’ size, shape, and internal structures (e.g., septa, solid tissue), aiding differentiation between benign and malignant tumors. However, the limited resolution of TVS may fail to detect small or early-stage tumors, and overlapping anatomical structures, such as bowel loops or normal ovarian tissue, complicate mass differentiation. Moreover, specific OC subtypes do not exhibit significant morphological changes in early phases, reducing sensitivity (Wu et al., 2018; Rosati et al., 2020).

Despite these limitations, TVS remains indispensable in the initial OC diagnosis due to accessibility and low cost. However, interpretation often varies significantly with radiologist experience, resulting in diagnostic inconsistencies and clinical errors (Bäumler et al., 2020; Nebgen et al., 2019). These issues underscore the critical need for standardized, objective, and automated diagnostic methods that enhance accuracy and reduce inter-observer variability.

Artificial intelligence (AI) integration has significantly advanced medical imaging diagnostics, improving tumor identification accuracy and consistency. Convolutional neural networks (CNNs), a sophisticated deep learning (DL) architecture, have demonstrated over 90% accuracy in extracting complex TVS image features and classifying ovarian malignancy in several studies (Akazawa and Hashimoto, 2021; Sone et al., 2021). Beyond accuracy, AI reduces human interpretation errors and enables the analysis of large datasets (Falana et al., 2023; Sahu and Shrivastava, 2023).

However, several challenges hinder the clinical translation of AI in OC detection. Crucially, existing DL models suffer from inadequate dataset representativeness, as most research uses datasets from single institutions, leading to population biases. AI model performance notably declines when tested on populations with different ethnic, geographic, or technological characteristics (Noseworthy et al., 2020). Additionally, heterogeneity in TVS image quality, formats, resolution, and acquisition protocols across institutions further impairs AI model reproducibility and generalizability (Raciti et al., 2023).

A significant barrier is the absence of robust prospective clinical validation. Most AI algorithms have been validated retrospectively, limiting insights into their real-time clinical applicability (Raciti et al., 2023). Furthermore, lack of standardized annotation and segmentation protocols significantly impacts AI model accuracy, with manual segmentation inconsistencies affecting performance by up to 20% (Heinlein et al., 2024; Ho et al., 2022). The absence of international consortia, standardized benchmarking, and sufficiently large, diverse, open-access TVS image databases also restrict the comparative evaluation and robust training of AI models. While initiatives like The Cancer Imaging Archive (TCIA) have addressed similar needs in other cancer areas, a parallel effort for OC is currently lacking (NIH, n.d.).

This systematic review and quantitative meta-analysis address these critical knowledge gaps by evaluating the diagnostic performance of AI models applied specifically to B-mode TVS images for early OC detection. Through a comprehensive comparison of accuracy, sensitivity, specificity, and area under the curve (AUC) across CNNs, classical machine learning algorithms, and transformer-based models, the study assesses how methodological factors, such as segmentation and dataset size, influence model performance. Clarifying these factors is expected to enhance clinical practice directly by guiding the development of robust, standardized AI tools capable of improving early OC diagnosis, thereby increasing patient survival rates and clinical outcomes.

## 2 Methodology

This study was designed and conducted following the PRISMA 2020 guidelines (Preferred Reporting Items for Systematic Reviews and Meta-Analyses) (Liberati et al., 2009; Page et al., 2021) to ensure transparency, reproducibility, and comprehensiveness in the systematic review and meta-analysis (Supplementary Tables S1, S2). The methodological protocol included a predefined search strategy, explicit eligibility criteria, risk of bias assessment, and statistical analysis of the extracted data. Specifically, the systematic review was guided by the following research questions:

- What is the diagnostic accuracy (accuracy, sensitivity, specificity, and AUC) of AI-based models for ovarian mass classification using B-mode ultrasound?
- Which methodological factors, such as segmentation methodology (automatic vs. manual), dataset size, AI model architecture, and risk of bias significantly influence the diagnostic performance of AI models?

### 2.1 Search strategy

In January 2025, a comprehensive search was conducted in three high-impact scientific databases: PubMed, IEEE Xplore, and Scopus. The search strategy included the following terms combined using Boolean operators: (“machine learning” OR “artificial intelligence” OR “deep learning” OR “neural network”) AND (“ovarian cancer” OR “ovarian tumor”) AND “ultrasound.” No language or publication type restrictions were applied during the initial search.

### 2.2 Eligibility criteria

The following inclusion and exclusion criteria were explicitly defined to ensure transparency and reproducibility in the systematic selection of studies (see Table 1).

**Table 1:** Inclusion and exclusion criteria for study selection.

Inclusion criteria	Exclusion criteria
Original research articles evaluating AI models	Systematic reviews, meta-analyses, letters to the editor, or abstracts from conferences
Studies utilizing 2D B-mode transvaginal ultrasound (TVS) images	Studies employing imaging modalities other than ultrasound (e.g., CT, MRI)
Studies involving the detection or classification of ovarian cancer in humans	Studies exclusively focused on serological biomarkers, genomic analyses, or animal models
Studies reporting at least one performance metric (accuracy, sensitivity, specificity, or AUC)	Studies without accessible full text or not reporting any relevant performance metric
Studies based on real patient data	Purely theoretical studies without clinical validation
Full-text articles published in English or Spanish	Studies published in languages other than English or Spanish
Studies published up to December 2024	Studies published after December 2024

Studies meeting all inclusion criteria and none of the exclusion criteria were eligible for inclusion in this systematic review and meta-analysis.

### 2.3 Study selection process

Two independent reviewers (IGA and FVF) initially evaluated the title and abstract of each article identified through the database search, applying the inclusion and exclusion criteria explicitly defined in Section 2.2. This preliminary assessment allowed for the exclusion of clearly irrelevant or ineligible studies. In cases of discrepancies during this initial stage, a third reviewer (EDB) was consulted to reach consensus. Subsequently, the full texts of the preselected articles were reviewed again by both reviewers (IGA and FVF) to confirm their definitive eligibility for inclusion in the quantitative analysis.

### 2.4 Data extraction

The following variables were extracted from each study: author, year, type of segmentation, model architecture, model name, image dataset size, type of ovarian masses, number of classes, and performance metrics (accuracy, sensitivity, specificity, and AUC). For studies reporting multiple models, the one with the best overall performance was selected to avoid data duplication. The information was systematized into a structured database for subsequent statistical analysis.

### 2.5 Risk of bias assessment

The methodological quality of the included studies was assessed using the PROBAST tool (Prediction model Risk of Bias Assessment Tool) (Wolff et al., 2019), which evaluates the risk of bias and applicability in studies that develop or validate prediction models. PROBAST comprises 20 items grouped into four domains: (i) participant selection, (ii) predictors, (iii) outcomes, and (iv) statistical analysis. Two reviewers (IGA and FVF) independently performed this evaluation. Discrepancies were resolved by consensus. A detailed assessment of the 20 PROBAST items for each study and domain-specific classifications is included as Supplementary Tables S3, S4.

### 2.6 Statistical analysis

#### 2.6.1 Descriptive statistics and distribution assessment

Descriptive statistics were calculated for performance metrics, including accuracy, sensitivity, specificity, and AUC, and are reported as means, standard deviations, and ranges. The Shapiro–Wilk test was used to assess the normality of distributions, while Levene’s test was applied to evaluate the homogeneity of variances.

Because F1-score was rarely reported and often lacked the underlying confusion matrix, we did not meta-analyze F1. For interpretability, accuracy, sensitivity, specificity, and AUC remained our primary endpoints.

#### 2.6.2 Comparison between segmentation methods (automatic vs. manual)

Given the presence of non-normal distributions and limited subgroup sizes, non-parametric tests were prioritized to enhance statistical validity. Specifically, a Mann–Whitney U test was used to compare accuracy between automatic and manual segmentation strategies.

#### 2.6.3 Comparison across AI model architectures

Differences in accuracy across AI architecture categories (e.g., CNN, ML, ANN) were assessed using a Kruskal–Wallis H test. Additionally, performance variation between DL models (e.g., CNNs) and classical machine learning approaches was evaluated using one-way analysis of variance (ANOVA).

#### 2.6.4 Correlation between dataset size and diagnostic performance

The relationship between dataset size and diagnostic performance was explored using Pearson’s correlation, excluding studies with more than 5,000 images to mitigate the influence of extreme values.

#### 2.6.5 Meta-regression analysis of methodological factors

A meta-regression analysis was performed using ordinary least squares (OLS) modeling to investigate the combined influence of methodological variables on diagnostic performance. Accuracy was modeled as the dependent variable, and key predictors included dataset size, segmentation type (automatic vs. manual), model architecture (CNN vs. other), and risk of bias (high vs. low). The regression included 26 studies with complete data and demonstrated that segmentation type was a significant predictor of accuracy (*β* = 0.0656, *p* = 0.007), while the other covariates did not reach statistical significance. The model explained approximately 32% of the variance in accuracy (adjusted *R*^2^ = 0.32), supporting the relevance of segmentation quality as a determinant of AI model performance.

#### 2.6.6 Subgroup and sensitivity analyses

Subgroup analyses were conducted based on risk of bias (assessed by PROBAST), and a sensitivity analysis was performed by excluding studies rated as high risk to determine the robustness of findings.

#### 2.6.7 Software and reproducibility

All statistical analyses were conducted using Python (v3.12), leveraging the pandas, numpy, scipy, statsmodels, matplotlib, and seaborn libraries. Complete analysis code and data visualizations are available upon request.

All figures include concise alternative text in the captions, and a separate Supplementary material provides long textual descriptions.

## 3 Results

**Figure 1:**
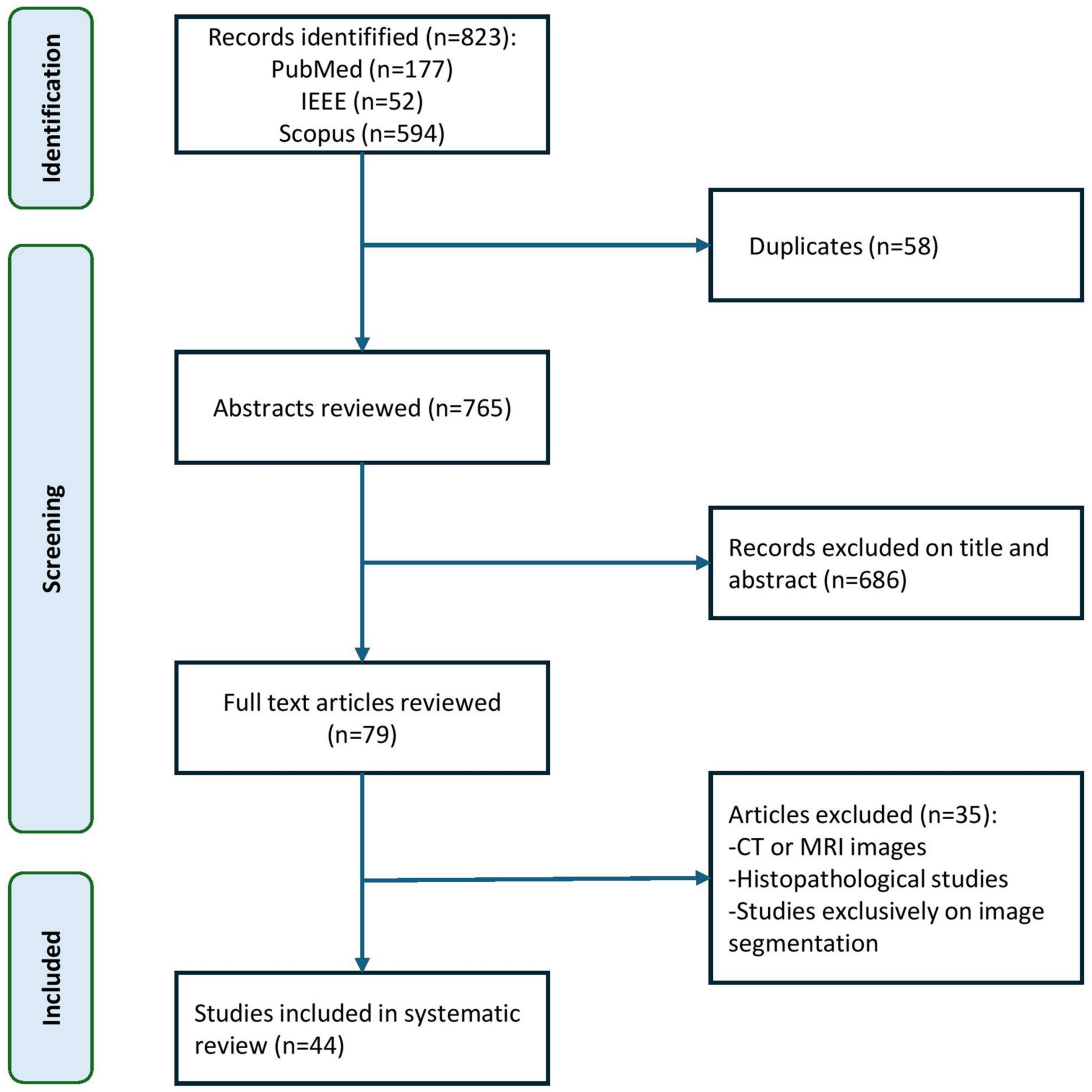
PRISMA flow diagram of our study. The figure illustrates the study selection process following PRISMA 2020 guidelines. A total of 823 records were identified through three databases (PubMed, IEEE Xplore, and Scopus). After removing 58 duplicates, 765 titles and abstracts were screened, excluding 686 studies that did not meet the eligibility criteria. Seventy-nine full-text articles were assessed for inclusion, of which 35 were excluded for using non-ultrasound imaging modalities (e.g., CT or MRI), focusing exclusively on segmentation methods, or lacking histopathological validation. Ultimately, 44 studies were included in the final quantitative analysis.

The systematic search in the PubMed, IEEE Xplore, and Scopus databases yielded 823 studies. After removing 58 duplicates, 765 titles and abstracts were screened. Of these, 686 were excluded for not meeting the inclusion criteria, resulting in 79 articles for full-text review. Finally, 44 studies were included in the quantitative analysis (Figure 1).

The studies cover a period up to December 2024. Collectively, they analyzed over 650,000 B-mode TVS images for ovarian mass classification using various AI models. Most studies (*n* = 27; 61.4%) used automatic segmentation, while the remainder (*n* = 17; 38.6%) employed manual segmentation. The predominant architectures were CNNs, followed by classical ML algorithms, conventional artificial neural networks (ANNs), and transformer-based architectures that have emerged in recent years (Table 2).

**Table 2:** List of the 44 articles analyzed.

Id	References	Year	Segmentation	Algorithm architecture	Artificial intelligence model	Size of dataset	Type of masses	Classes	Accuracy	Sensitivity	Specificity	AUC	Risk of bias
1	Ravishankar et al. (2023)	2023	Automatic	CNN	OCD-FCNN	440	Cysts	8	0.984	0.97	–	–	High
2	Li et al. (2022)	2022	Automatic	CNN	LKResNet-18	5,714	Tumors	3	0.9145	0.918	0.918	–	Low
3	Fan et al. (2023)	2023	Automatic	CNN	Ocys-Net	750	Cysts	3	0.955	–	–	0.885	Unclear
4	Al-karawi et al. (2021)	2021	Manual	ML	SVM	242	Tumors	3	0.8058	0.8104	0.8022	–	Low
5	Patil et al. (2024)	2024	Automatic	ML	RF	187	Tumors	3	0.86	–	–	–	Unclear
6	Kiruthika et al. (2023)	2023	Automatic	ML	SVM	630	Tumors	3	0.965	0.96	0.955	–	Low
7	Wang et al. (2021)	2021	Manual	CNN	ResNet-34	279	Tumors	3	0.914	0.914	0.914	0.963	Low
8	Shih-Tien et al. (2022)	2022	Automatic	ML	EL	1896	Tumors	2	0.9215	0.9137	0.9292	–	Low
9	Meijing et al. (2023)	2023	Manual	CNN	ResNext50	1,142	Cysts	7	0.952	0.895	0.992	0.997	Low
10	Chen et al. (2022)	2022	Manual	CNN	ResNet-18	422	Tumors	2	–	0.92	0.85	0.93	Low
11	Gao et al. (2022)	2022	Automatic	CNN	DenseNet-121	575,930	Tumors	2	0.888	0.789	0.932	0.911	Low
12	Xiang et al. (2024)	2024	Automatic	ML	EL	3,972	Tumors	2	0.876	0.973	0.741	0.97	Low
13	Du et al. (2024)	2024	Manual	ANN	DLRN	849	Tumors	2	0.871	0.733	0.880	0.928	Unclear
14	Miao et al. (2024)	2024	Automatic	CNN	ConvNeXt	575	Cysts	2	0.90	0.90	–	0.90	Unclear
15	Alwan et al. (2023)	2023	Automatic	CNN	CNN	196	Tumors	2	0.9897	–	–	–	High
16	Martínez-Más et al. (2019)	2019	Manual	ML	SVM	187	Tumors	2	0.8770	0.91	0.83	0.8740	Unclear
17	Acharya et al. (2014)	2014	Automatic	ANN	PNN	2,600	Tumors	2	0.9981	0.9992	0.9969	–	High
18	Hussein et al. (2020)	2020	Automatic	FDA	Viola-Jones	125	Tumors	2	0.9484	0.9696	0.9032	–	Unclear
19	Hussein et al. (2022)	2021	Automatic	ANN	ANN	250	Tumors	2	0.9587	0.9701	0.9333	–	Unclear
20	Acharya et al. (2014)	2014	Automatic	ANN	PNN	2,600	Tumors	2	1.00	1.00	1.00	–	High
21	Jeevitha and Priya (2022)	2022	Automatic	ML	SVM	100	Cysts	3	0.985	0.940	–	–	Unclear
22	Wang et al. (2024)	2024	Manual	CNN	ResNet-50	1,054	Tumors	2	0.9476	0.9428	0.9500	0.984	Low
23	Narmatha et al. (2023)	2023	Automatic	RNN	Deep Q-Network	478	Cysts	7	0.96	0.96	–	–	Unclear
24	Yuyeon et al. (2022)	2022	Automatic	CNN	DenseNet161	1,613	Cysts	5	0.9012	0.8667	0.9185	0.9406	Low
25	Pham and Le (2024)	2024	Automatic	CNN	YOLOv8	1,469	Tumors	8	0.9126	0.8330	–	–	Low
26	Kongara et al. (2024)	2024	Automatic	CNN	CNN	3,280	Cysts	2	0.9918	–	–	–	Unclear
27	Li et al. (2024)	2024	Automatic	CNN	PMFFNet	1,469	Cysts	7	0.9724	0.9855	–	–	Low
28	Miao et al. (2023)	2023	Automatic	CNN	ResNet-34	1,130	Tumors	2	–	0.97	0.93	0.95	Low
29	Moro et al. (2024)	2024	Manual	ML	RF	775	Tumors	2	–	0.99	0.64	0.88	Low
30	Chiappa et al. (2021)	2020	Manual	CNN	CNN	241	Cysts	3	0.83	0.78	0.85	0.88	Low
31	Xi et al. (2023)	2023	Automatic	CNN	DenseNet	1,103	Tumors	2	0.964	0.997	0.952	0.973	Low
32	Ștefan et al. (2021)	2021	Automatic	ML	KNN	123	Tumors	2	–	0.9048	0.931	0.951	Unclear
33	Christiansen et al. (2021)	2021	Manual	ML	EL	3,077	Tumors	2	–	0.971	0.937	0.958	Low
34	Aramendía-Vidaurreta et al. (2015)	2015	Manual	ANN	MLP	145	Tumors	2	0.9878	0.9850	0.9890	0.997	Unclear
35	Liu et al. (2024)	2024	Manual	CNN	ResNet-101	1,080	Tumors	2	0.849	0.930	0.817	0.935	Low
36	Liu et al. (2024)	2024	Manual	ML	LR	407	Cysts	2	0.948	0.955	0.942	0.981	Low
37	Du et al. (2024)	2024	Manual	CNN	ResNet-50	849	Tumors	3	0.8003	0.7515	–	0.85	Low
38	Tang et al. (2022)	2022	Manual	ML	LR	206	Tumors	2	–	–	–	0.886	Low
39	Acharya et al. (2018)	2018	Manual	ML	RF	469	Tumors	2	0.8060	0.8140	0.7630	–	High
40	Sha (2024)	2024	Automatic	CNN	AdaResU-Net	700	Tumors	2	0.9887	0.9850	0.9960	–	High
41	Xie et al. (2024)	2024	Automatic	CNN	YOLOv8	1,619	Tumors	2	0.935	0.905	0.935	0.930	Low
42	Giourga et al. (2024)	2024	Automatic	ML	EL	3,510	Cysts	2	0.909	0.965	0.881	0.922	Low
43	He et al. (2024)	2024	Manual	TBM	Swin transformer	7,639	Tumors	2	–	0.872	0.943	0.920	Low
44	Dai et al. (2024)	2024	Automatic	TBM	Pyramid visual transformer	6,938	Tumors	3	0.873	0.878	0.869	0.941	Low

AUC, area under the curve; CNN, convolutional neural network; FCNN, fuzzy rule-based convolutional neural network; ML, machine learning; SVM, support vector machine: RF, random forest; EL, ensemble learning; ANN, artificial neural network; DLRN, deep learning radiomics nomogram; PNN, probabilistic neural network; FDA, face detection algorithm; RNN, recurrent neural network; KNN, K-nearest neighbor; MLP, multilayer perceptron networks; LR, logistic regression; TBM, transformer-based models.

### 3.1 Overall performance of AI models

The analysis of the 44 included studies revealed the high average diagnostic performance of AI models applied to B-mode TVS images for OC detection. The mean accuracy was 92.3% ± 5.8, with mean sensitivity and specificity of 91.6% ± 7.2 and 90.1% ± 8.1, respectively. AUC values were reported in only 23 studies, with a mean of 0.93 ± 0.04, reflecting strong overall discriminative capacity. However, the partial availability of AUC reporting may indicate a potential reporting bias that limits the robustness of comparative analysis across all models. Beyond AUC’s limited reporting (23/44 studies), F1-score was scarcely available across the corpus. This pattern likely reflects historical reliance on accuracy/sensitivity/specificity in ultrasound AI, frequent absence of continuous model scores (hindering AUC), and the lack of confusion matrices or class-wise results needed for F1. In addition, F1 is sometimes reported as Dice in segmentation studies; because our review targets classification performance, segmentation-specific Dice metrics were not pooled, which might also contribute to the perceived under-reporting of F1.

**Figure 2:**
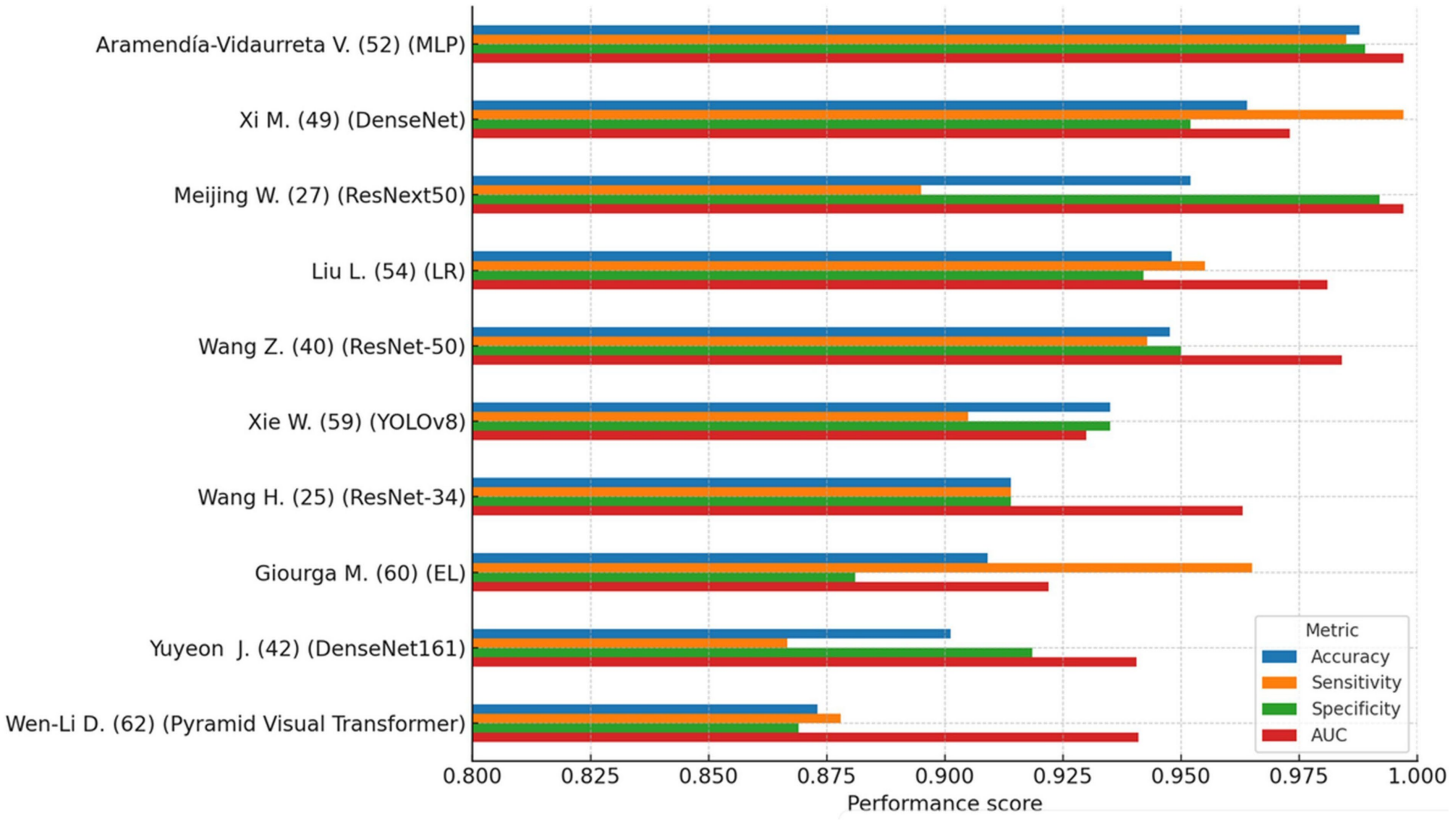
Top 10 AI models (comparison of accuracy, sensitivity, specificity, and AUC). This figure compares performance metrics (accuracy, sensitivity, specificity, and AUC) of the 10 highest-performing AI models identified in the review. Generally high accuracy is observed across models, but some exhibit trade-offs between sensitivity and specificity. This variability highlights the importance of selecting models not only with high overall accuracy but also clinically meaningful balance to minimize diagnostic errors, particularly false positives and false negatives.

Figure 2 provides a comparative overview of the four main performance metrics for the 10 top-performing models. This visualization highlights how specific models exhibit strong accuracy yet relatively lower specificity, an observation with important clinical implications when considering false-positive rates in diagnostic triage.

Several models, including those based on OCD-FCNN, probabilistic neural networks (PNN), and ResNet-34, reported peak performance values exceeding 95%. However, many of these models were trained and tested on small or non-external datasets, lacked proper cross-validation, or relied exclusively on internal test sets. Such methodological limitations increase the likelihood of overfitting and restrict the generalizability of reported outcomes. None of the highest-performing models reported prospective validation or integration into clinical workflows, which remains essential for evaluating real-world applicability.

To assess whether methodological design influenced diagnostic performance, non-parametric tests were conducted using accuracy as the outcome variable. A Mann–Whitney *U*-test revealed a statistically significant difference in accuracy between models using automatic versus manual segmentation (*U* = 234.0, *p* = 0.007), favoring automatic methods. This finding suggests that automated segmentation enhances standardization and reduces variability across studies.

**Figure 3:**
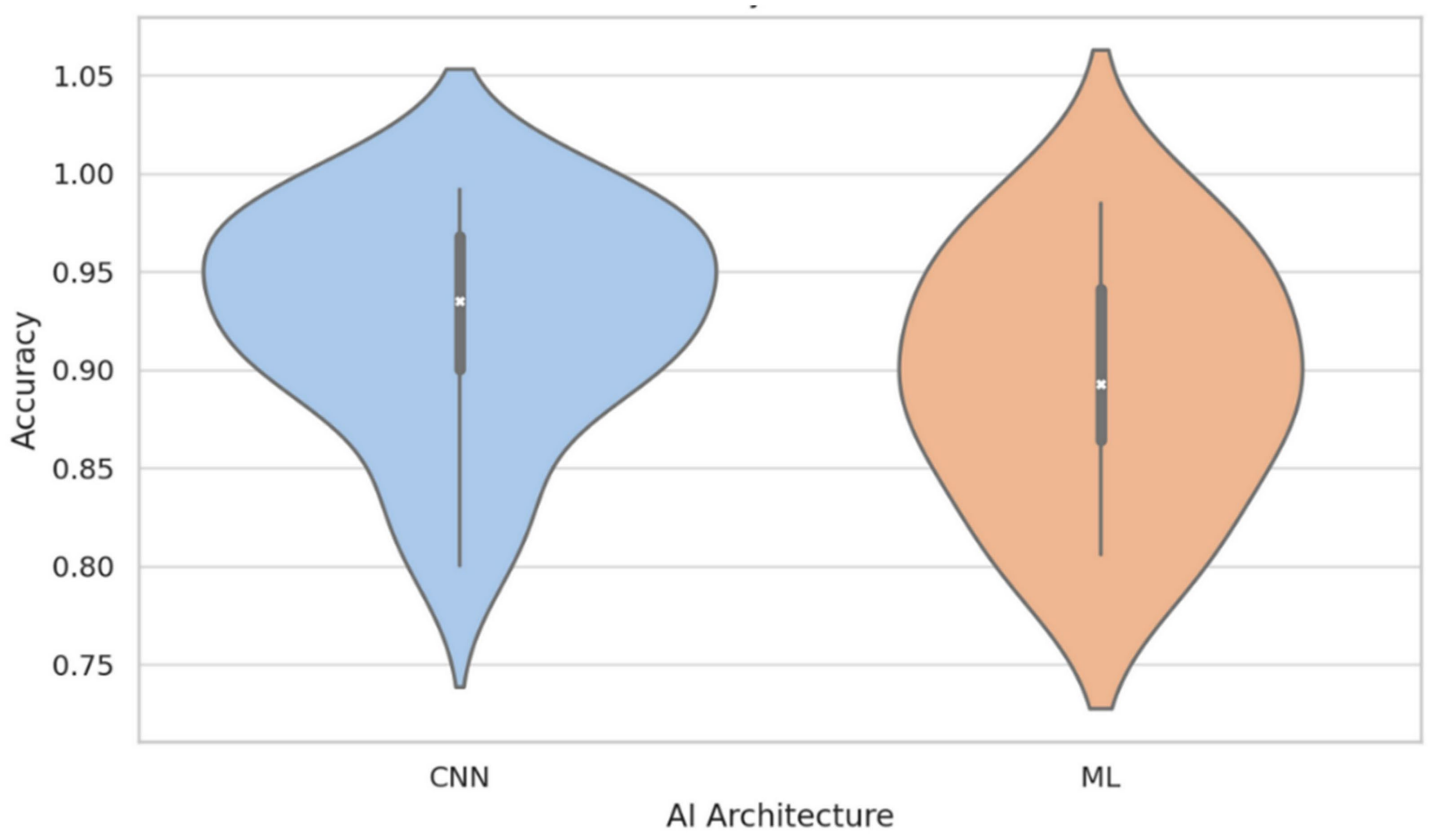
Accuracy distribution by AI architecture: CNN vs. classical machine learning (ML). This figure illustrates that, although there is no statistically significant difference between architectures (*p* = 0.258), CNN-based models tend to display higher median accuracy and reduced variability compared to classical ML models. This result suggests a potential advantage of CNNs, likely due to their superior ability to capture complex features from medical images, though methodological advances in recent studies may also contribute to this observed trend.

Conversely, a Kruskal–Wallis test comparing performance across AI architectures (e.g., CNN, ML, ANN) did not identify statistically significant differences (*H* = 6.53, *p* = 0.258), indicating that no specific architectural family demonstrated superior accuracy within the current dataset. Nevertheless, visual inspection using violin plots (Figure 3) showed a moderately higher central tendency and reduced the variance in accuracy among CNN-based models compared to classical machine learning (ML) approaches. While this pattern may reflect the architectural strengths of CNNs in capturing spatial hierarchies within medical images (Litjens et al., 2017), it should be interpreted cautiously. CNN-based models were more frequently applied in recent studies, which may also have benefited from advances in data augmentation, automatic segmentation, and preprocessing pipelines. Therefore, the observed trend could be confounded by methodological improvements rather than an inherent advantage of architecture.

It is also important to note that model performance was evaluated exclusively using accuracy, as this was the most consistently reported metric across studies. While this allowed for comparability, it may limit interpretability in class-imbalanced settings, where metrics such as AUC or F1-score are often more informative. Future studies should prioritize the reporting of multiple complementary metrics to capture diagnostic value more comprehensively.

These descriptive findings are further explored and formally tested in the meta-regression presented in Section 3.4.

In summary, while reported performance metrics are generally high, the absence of standardized validation protocols, partial reporting of key metrics, lack of weighted or stratified analyses, and underreporting of methodological variables (especially segmentation and validation strategies) limit the interpretability and clinical generalizability of the findings. Future studies should adopt harmonized reporting guidelines (e.g., TRIPOD-AI, PROBAST-AI), employ multicenter and external validation, and report performance metrics in clinically meaningful terms to support reliable and reproducible integration into diagnostic workflows.

### 3.2 Relationship between dataset size and performance

The relationship between the number of images used to train AI models and their diagnostic performance was evaluated using non-parametric correlation analysis. Although Pearson’s method was initially considered, the Shapiro–Wilk test confirmed that dataset size and performance metrics (accuracy, sensitivity, specificity) were not normally distributed (*p* < 0.001 for all), prompting the use of Spearman’s rank correlation.

Studies with more than 5,000 images were excluded from this analysis to reduce the risk of statistical distortion from highly imbalanced sample sizes. While large-scale datasets (up to 575,000 images) have become increasingly common in AI development, such volumes do not reflect typical clinical practice and may disproportionately drive correlation estimates. The 5,000-image threshold was selected to capture real-world data conditions better while preserving inter-study variability. Descriptive analysis of the full dataset showed that this threshold approximately corresponds to the 75th percentile of dataset sizes among included studies.

Spearman correlation coefficients between dataset size (≤5,000) and model performance metrics were weak and statistically non-significant. Specifically, the correlation with accuracy was *ρ* = 0.080 (*p* = 0.653), with a 95% confidence interval of −0.27 to 0.41 and an *R*^2^ of 0.006, suggesting that less than 1% of the variation in accuracy could be explained by dataset size. For sensitivity, the correlation was ρ = 0.246 (*p* = 0.154; 95% CI: −0.09 to 0.54; *R*^2^ = 0.061), and for specificity, ρ = 0.183 (*p* = 0.350; 95% CI: −0.20 to 0.52; *R*^2^ = 0.034). Table 3 summarizes these results, including the correlation coefficients, confidence intervals, and the proportion of explained variance.

**Table 3:** Correlation between dataset size and performance metrics.

Performance metric	Spearman ρ	95% CI	*R* ^2^
Accuracy	0.080	[−0.27, 0.41]	0.006
Sensitivity	0.246	[−0.09, 0.54]	0.061
Specificity	0.183	[−0.20, 0.52]	0.034

While these findings suggest that increasing dataset size within the studied range does not systematically improve model performance, this interpretation should be cautiously made. The exclusion of large datasets may limit the generalizability of these findings, and potential interaction effects, such as those involving the segmentation method, risk of bias, or model architecture, were not examined in this univariate analysis. These results are, however, consistent with the multivariable meta-regression analysis presented in Section 4, in which dataset size did not emerge as a significant independent predictor of accuracy.

One plausible explanation lies in the widespread adoption of data augmentation strategies. Techniques such as image rotation, scaling, contrast adjustment, and noise addition simulate data variability and may reduce the dependency on raw volume. However, excessive use of augmentation may also lead to redundancy or learning saturation, where additional data no longer meaningfully improves generalization.

This interpretation is in line with prior literature. For instance, Roberts et al. (2021) found that dataset size was not consistently associated with performance in a comprehensive medical imaging AI studies review. Instead, methodological rigor, validation strategy, and data diversity were identified as stronger predictors of performance. Furthermore, the risk of performance overestimation due to augmented or homogeneous datasets remains a critical concern in model evaluation.

**Figure 4:**
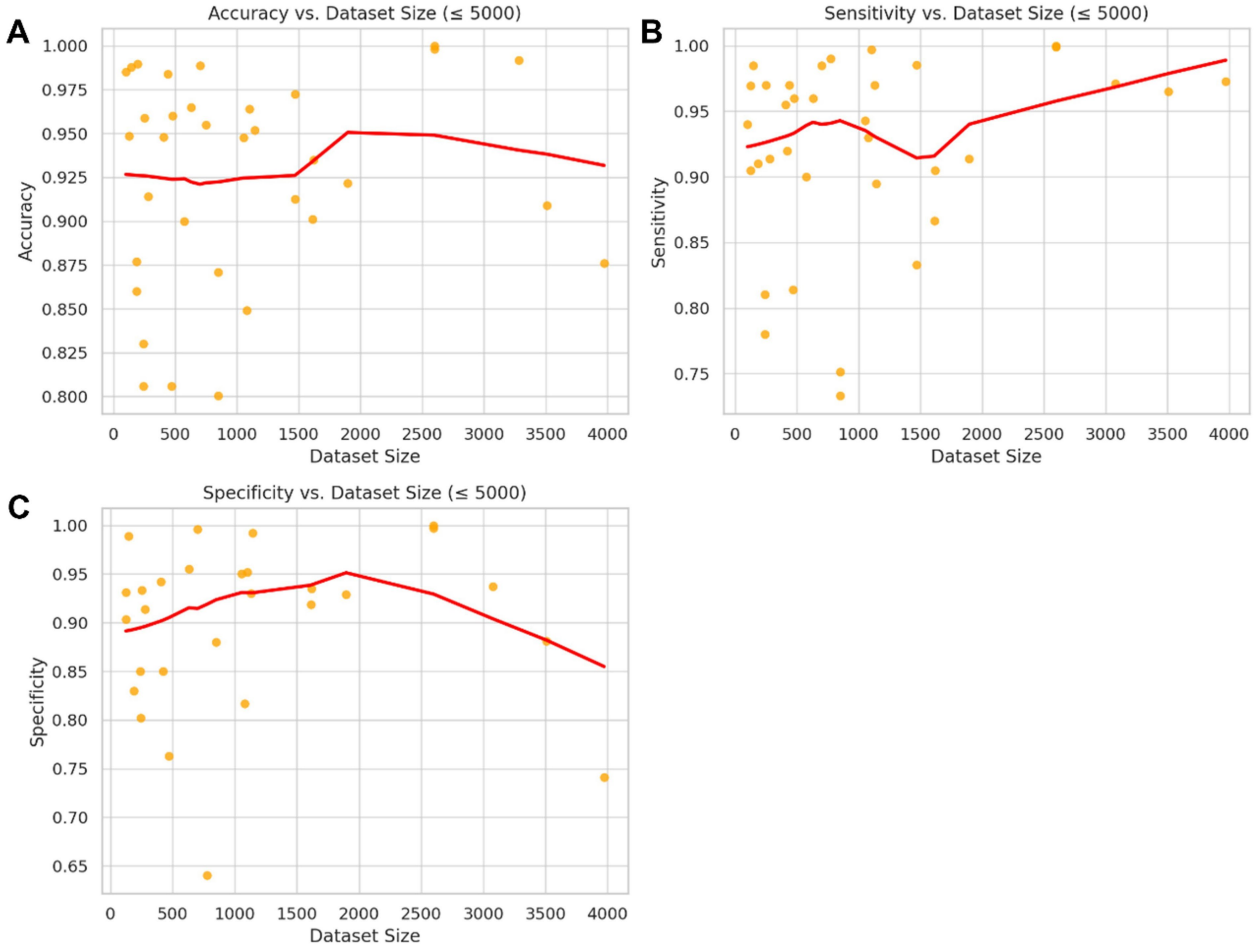
Non-parametric association between dataset size and model performance metrics (≤5,000 images). Scatter plots depict the relationship between dataset size and three key performance metrics: **(A)** accuracy, **(B)** sensitivity, and **(C)** specificity across studies with ≤5,000 training images. The red lines represent LOWESS (locally weighted scatterplot smoothing) regression fits with 95% confidence bands. Although minor local variations are observed, no clear or systematic trend indicates a significant improvement in these performance metrics with increasing dataset size within the clinically relevant range analyzed. This finding suggests that methodological factors other than dataset size may have a greater influence on diagnostic model accuracy.

Figure 4 presents scatterplots of accuracy, sensitivity, and specificity versus dataset size (≤5,000), each overlaid with a non-parametric LOWESS regression line and 95% confidence bands. While substantial scatters are observed across all metrics, the absence of clear or consistent directional trends underscores the importance of factors beyond sample size, such as annotation quality and experimental design in developing reliable diagnostic models.

### 3.3 Comparison between automatic and manual segmentation

To evaluate the impact of segmentation strategy on the diagnostic performance of AI models, performance metrics were compared between studies that implemented automatic segmentation (*n* = 27) and those that used manual segmentation (*n* = 17).

Models using automatic segmentation achieved a significantly higher average accuracy (94.2% ± 4.3) than manual segmentation (88.2% ± 6.6, *p* = 0.012). Sensitivity also favored automatic segmentation (93.7% ± 5.6 vs. 88.6% ± 8.3, *p* = 0.042). Although specificity was higher in the automatic group (92.5% ± 6.0 vs. 87.3% ± 9.5), the difference was not statistically significant (*p* = 0.084). AUC values were nearly identical between both groups (*p* = 0.839). Confidence intervals for these comparisons were not reported but are recommended for future studies to enhance the interpretability and reproducibility of statistical estimates.

Levene’s test revealed a significantly more significant variance in specificity within the manual segmentation group (*p* = 0.045), indicating less consistency. This is consistent with previous literature findings, highlighting manual segmentation’s susceptibility to inter- and intra-observer variability, particularly when standardized annotation protocols or multiple expert raters are not employed (Taha and Hanbury, 2015; Menze et al., 2015).

Although the primary studies reported heterogeneous segmentation details that precluded a stratified meta-analysis by architecture, a brief practical comparison is informative for clinical implementation. U-Net remains the canonical encoder–decoder with skip connections that performs well when lesion boundaries are reasonably defined, and training data are limited. AdaResU-Net augments U-Net with residual blocks and adaptive mechanisms that enlarge the effective receptive field and stabilize gradient flow, improving boundary delineation in speckle-rich ultrasound and in the presence of heterogeneous echotexture. In practice, U-Net offers simplicity and fast deployment; AdaResU-Net can yield crisper contours and fewer leakage errors near cyst walls at the cost of extra parameters. These architectural tradeoffs are likely to contribute to the higher and less variable accuracy we observed with automated vs. manual segmentation. Future primary studies should report standardized segmentation metrics (e.g., Dice, surface distance) alongside classification endpoints to enable formal architecture-level synthesis.

**Figure 5:**
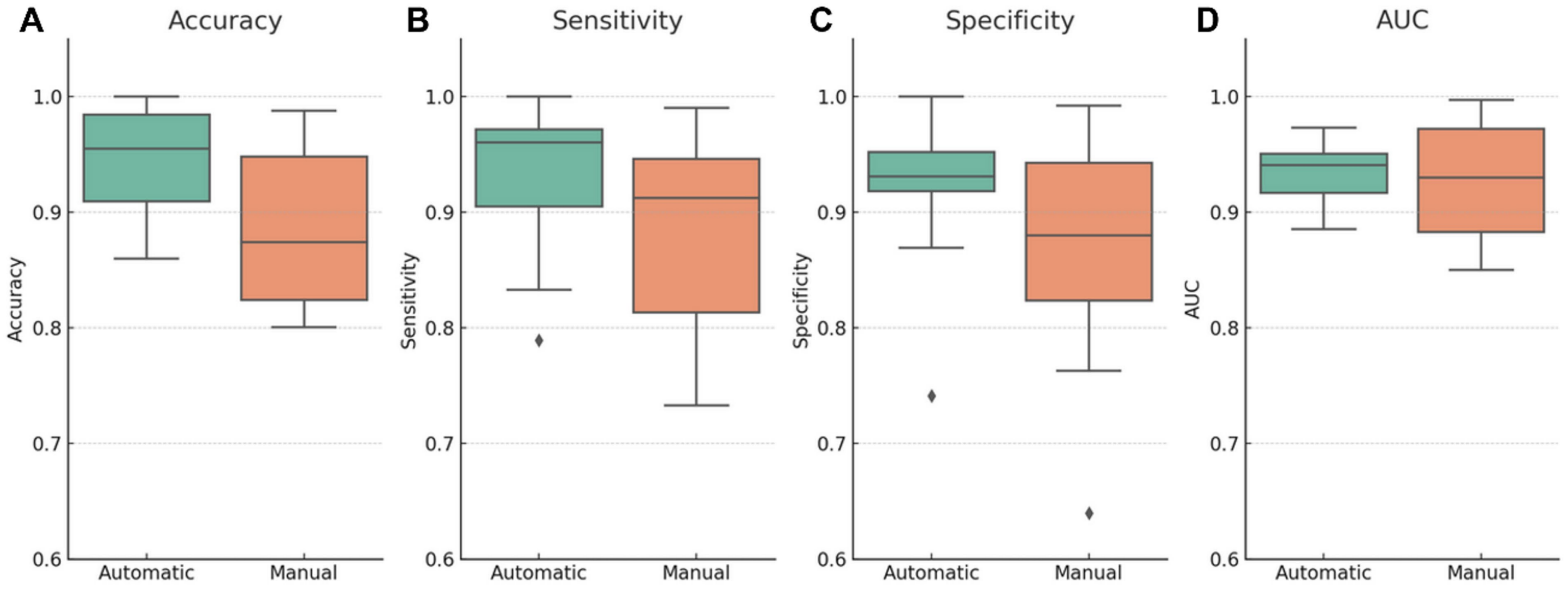
Diagnostic performance metrics stratified by segmentation type (automatic vs. manual). Boxplots illustrate the distribution of **(A)** accuracy, **(B)** sensitivity, **(C)** specificity, and **(D)** AUC across studies using either automatic or manual segmentation. Automatic segmentation models demonstrate higher mean values and reduced dispersion for most metrics, particularly accuracy and specificity. The manual segmentation group shows greater variability and outliers, suggesting less consistency. These visual differences align with the hypothesis that automated segmentation enhances reproducibility and standardization; however, causal interpretation should be made cautiously due to potential confounding factors.

Figure 5 presents comparative boxplots of accuracy, sensitivity, specificity, and AUC by segmentation type. The distributions reveal higher mean values for automatic segmentation across most metrics, lower dispersion, and fewer outliers, especially for specificity and sensitivity. This visual trend suggests increased consistency, which may be attributed to the standardization benefits of automated pipelines.

Table 4 provides a detailed summary of methodological characteristics and performance metrics stratified by segmentation type. Studies using automatic segmentation not only performed better on average but also used considerably larger datasets (mean = 22,941 vs. 1,121 images) and demonstrated lower standard deviation across metrics such as accuracy (4% vs. 7%) and specificity (6% vs. 10%).

**Table 4:** Dataset size and performance by segmentation type.

Segmentation	Size of dataset (mean)	Size of dataset (std)	Accuracy (mean)	Accuracy (std)	Sensitivity (mean)	Sensitivity (std)
Automatic	22940.63	110529.7	0.94	0.04	0.94	0.06
Manual	1121.35	1818.76	0.88	0.07	0.89	0.08

However, this interpretation should be approached with caution. Although these group-level comparisons suggest superior performance with automatic segmentation, the analysis did not control for potential confounders such as dataset size, model architecture, training methodology, or publication year. Importantly, these variables may co-vary with segmentation strategy, particularly since automatic methods are more prevalent in recent, technically advanced studies.

As shown in the meta-regression (Section 3.4), the segmentation strategy remained a significant independent predictor of accuracy even after adjusting for these covariates. Nevertheless, the unadjusted differences observed here might still reflect broader methodological convergence rather than a causal advantage of automatic segmentation per se.

Future studies should incorporate multivariable models, harmonized annotation protocols, and prospective designs to clarify the segmentation method’s isolated effect on AI model performance. Moreover, reporting standards such as STARD-AI and TRIPOD-AI should be adopted to ensure replicability and transparency in performance evaluation across studies (Collins et al., 2021; Sounderajah et al., 2021).

### 3.4 Meta-regression analysis of methodological factors

**Figure 6:**
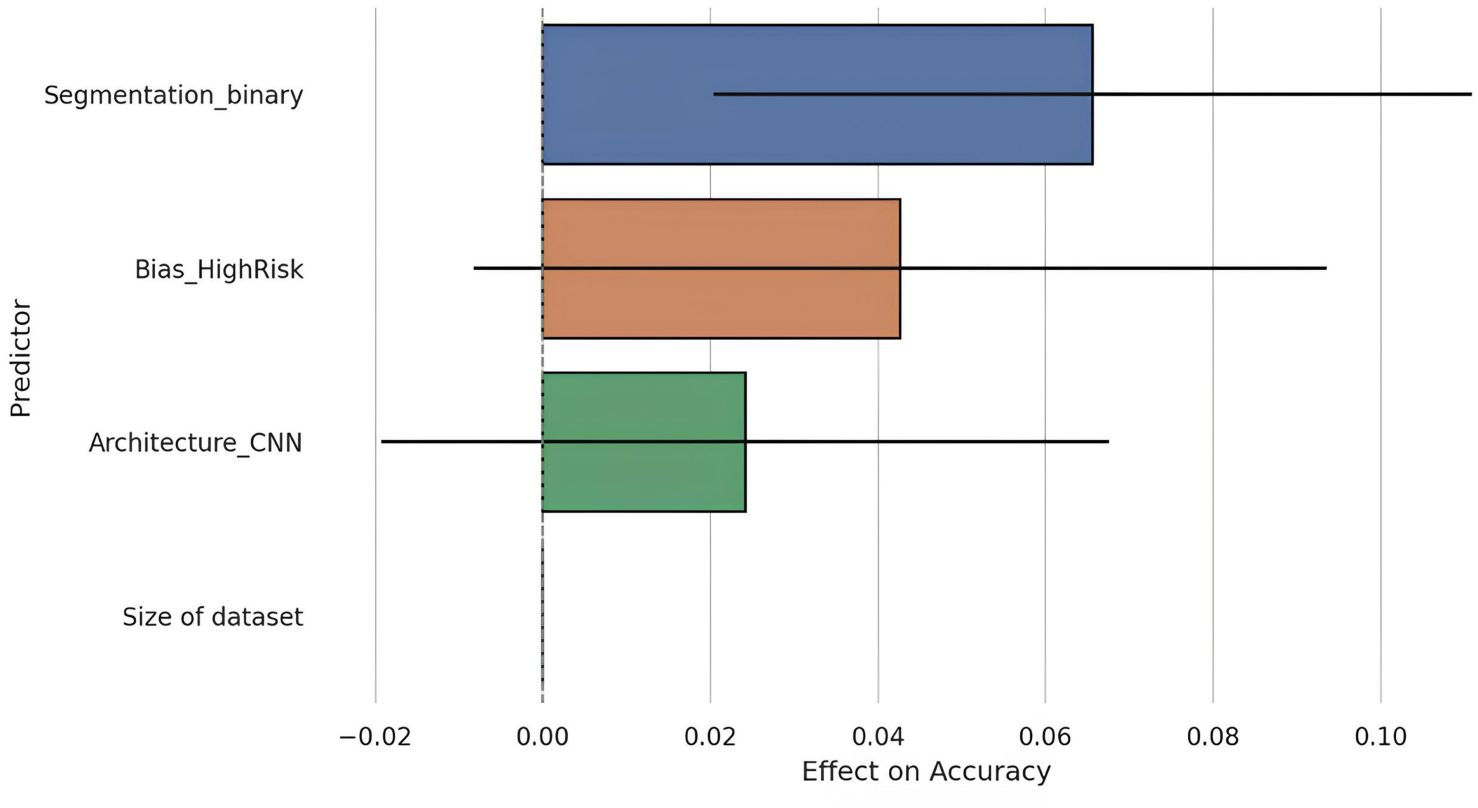
Meta-regression coefficients with 95% confidence intervals. This figure presents the coefficients resulting from the meta-regression analysis evaluating the relative influence of various methodological factors on diagnostic accuracy. Only automatic segmentation demonstrated a statistically significant effect on accuracy (*p* = 0.007), whereas other factors such as dataset size, architecture type (CNN vs. others), and risk of bias did not reach statistical significance. This outcome emphasizes the critical importance of automated segmentation quality as a key determinant of AI model performance.

To further explore the drivers of diagnostic performance, a meta-regression was performed using accuracy as the dependent variable and four predictors: dataset size, segmentation strategy (automatic vs. manual), model architecture (CNN vs. other), and risk of bias (high vs. low). The regression model included 26 studies with complete data across all variables (Figure 6).

The overall model was statistically significant (*F* = 3.98, *p* = 0.015), with an adjusted *R*^2^ of 0.32, indicating that the included predictors could explain approximately 32% of the variance in reported accuracy.

Among the covariates, segmentation strategy emerged as a significant predictor: studies using automatic segmentation reported on average, a 6.6 percentage point higher accuracy compared to those using manual segmentation (*β* = 0.0656, *p* = 0.007). This aligns with previous findings suggesting that automated preprocessing may reduce inter-observer variability and improve reproducibility.

Other predictors, such as dataset size, CNN architecture, and risk of bias, were not statistically significant at the conventional threshold (*p* > 0.050). However, the effect of high risk of bias approached significance (*β* = 0.0427, *p* = 0.096), suggesting a possible inflation of performance estimates in studies with methodological limitations.

Notably, dataset size was not a significant predictor (*p* = 0.323), corroborating earlier findings that performance does not linearly scale with sample size within the studied range, possibly due to saturation effects or compensatory use of data augmentation techniques.

These results reinforce the critical role of segmentation quality in shaping model performance and highlight the need for more standardized methodologies and transparent reporting in AI-based diagnostic research.

### 3.5 Temporal analysis

The progression of AI architectures in the included studies reflects a clear methodological shift over time. Temporal analysis of architectural usage revealed a transition from traditional ML techniques and ANNs to DL approaches, particularly CNNs. Between 2014 and 2018, studies primarily employed ML methods such as support vector machines (SVM), random forests (RF), and logistic regression (LR), representing approximately 85% of the methodologies used during this period. ANN-type architecture was also present, constituting roughly 15% of studies, while no CNN-based models were recorded before 2020.

**Figure 7:**
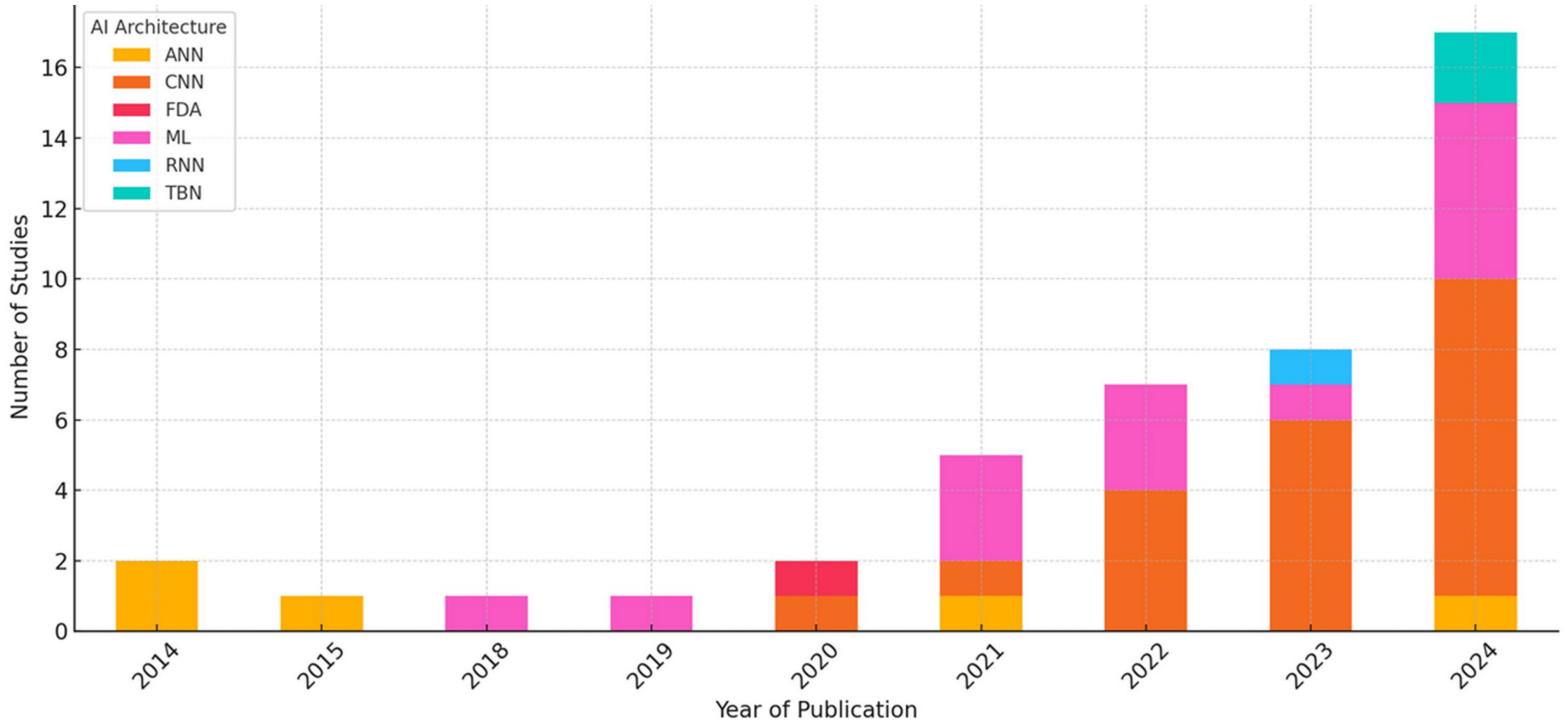
This figure illustrates the temporal evolution in the use of different AI architectures from 2014 to 2024. A clear shift is observed from classical machine learning techniques (ML and ANN) to deep learning models (CNN), particularly from 2021 onwards, with transformer-based models appearing more recently (2024). This evolution reflects a continuous methodological transition toward increasingly sophisticated architectures capable of directly and deeply extracting information from images. However, this shift may also coincide with general methodological improvements over time.

Figure 7 presents a stacked bar chart showing the number of studies using each architecture per year. CNNs emerged in 2020, accounting for 20% of the methodologies that year, and showed a marked increase in 2021, becoming the predominant architecture (65% of studies) in 2022. This trend intensified through 2023 (72% of studies) and 2024 (78% of studies), with CNNs accounting for more than half of the models evaluated annually. Transformer-based architectures, specifically Swin Transformer and Pyramid Vision Transformer, appeared exclusively in 2024, accounting for approximately 10% of the methodologies that year, indicating the beginning of a new phase of exploration focused on models with advanced contextual attention mechanisms and long-range feature integration.

This shift mirrors broader trends observed across diagnostic imaging AI, where deep architectures have largely replaced classical ML techniques due to their ability to learn hierarchical features directly from raw images without manual feature engineering (Litjens et al., 2017; Esteva et al., 2019). However, this evolution may also explain some of the performance differences observed in earlier sections. For example, the predominance of CNNs in recent years may co-occur with advances in preprocessing, data augmentation, and training infrastructure, confounding the interpretation of architecture-based performance gains.

Notably, this trend may influence perceived model superiority, as CNN-based studies often reflect newer methodological standards, including automatic segmentation and more rigorous evaluation protocols. These temporal patterns underscore the importance of accounting for publication year and technological maturity when comparing performance across architectures or studies.

### 3.6 Heterogeneity and risk of bias analysis

**Figure 8:**
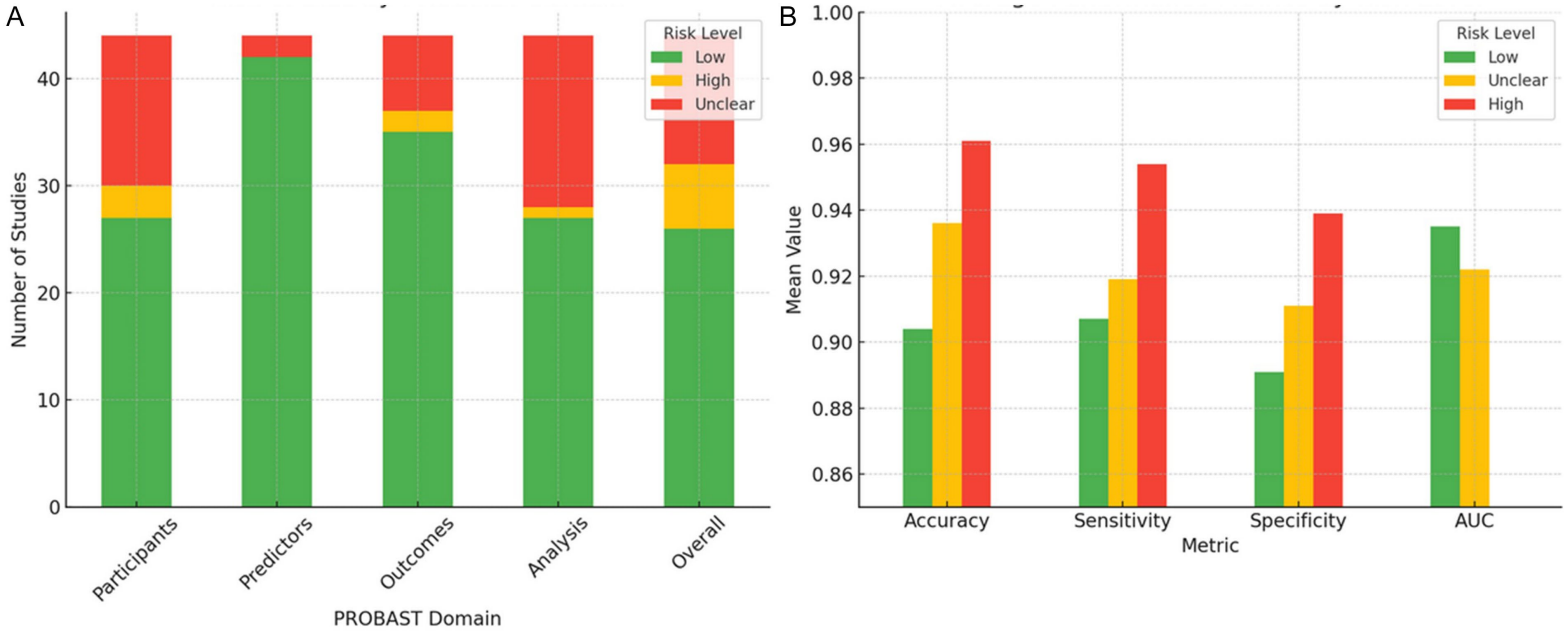
Distribution of risk of bias across PROBAST domains and associated model performance metrics. **(A)** Distribution of the 44 included studies across the four PROBAST domains (participants, predictors, outcomes, and analysis), categorized by overall risk of bias (low, unclear, and high). **(B)** Mean values of accuracy, sensitivity, specificity, and AUC, stratified by overall risk of bias. Studies classified as high risk consistently report higher mean values for accuracy, sensitivity, and specificity but exhibit greater variability and frequently lack AUC reporting, indicating potential methodological overfitting. Studies with low risk exhibit more consistent and reliable performance metrics.

To evaluate how methodological quality influences the reported performance of AI models, the 44 included studies were classified according to their overall risk of bias using the PROBAST tool. In total, 26 studies were classified as low risk of bias, 12 as unclear risk, and six as high risk (Figure 8). However, not all studies reported all performance metrics, and the number of studies included varied substantially by metric. This heterogeneity in reporting introduces selection bias and impairs comparability.

Studies classified as high risk of bias showed markedly elevated performance metrics, with a mean accuracy of 96.1% ± 7.6, a sensitivity of 95.4% ± 7.9, and a specificity of 93.9% ± 11.7. However, none of these studies reported areas under the curve (AUC) values, precluding the complete evaluation of discriminative performance. Moreover, the elevated standard deviation in specificity suggests potential overfitting, likely arising from methodologically weak practices such as internal validation without cross-validation, small sample sizes, and operator-dependent manual segmentation (Wolff et al., 2019; Collins et al., 2021). These practices have been consistently linked to inflated model performance in machine learning for medical imaging (Kelly et al., 2019).

In contrast, low-risk studies showed more conservative but consistent performance metrics, with lower dispersion and complete reporting of AUC. Specifically, they reported an accuracy of 90.4% ± 5.2 (*n* = 20), a sensitivity of 90.7% ± 7.0 (*n* = 25), a specificity of 89.1% ± 8.2 (*n* = 22), and an AUC of 93.5% ± 3.9 (*n* = 20). While these results appear numerically inferior, the reduced variability and broader metric completeness suggest higher methodological reliability and clinical applicability.

The unclear-risk group, often the result of poor reporting rather than clearly defined methodological shortcomings, yielded intermediate metrics (accuracy: 93.6% ± 5.0; sensitivity: 91.9% ± 7.7; specificity: 91.1% ± 5.4; AUC: 92.2% ± 4.6). However, the small and variable sample sizes for each metric (e.g., *n* = 6 for specificity) compromise interpretability and hinder statistical power.

A sensitivity analysis was conducted excluding high-risk studies, which resulted in a notable reduction in extreme values (e.g., 100% accuracy) and decreased overall dispersion, particularly in specificity. However, this analysis was limited by the lack of formal statistical testing (e.g., ANOVA or Kruskal–Wallis) to assess whether differences between groups were statistically significant. Furthermore, no regression adjustment was made for potential confounders such as dataset size or model complexity. These omissions limit the strength of causal inferences between risk of bias and reported model performance.

The primary sources of methodological heterogeneity across studies were identified as follows: (i) reliance on manual, operator-dependent image segmentation; (ii) absence of cross-validation or external validation; (iii) small sample sizes (<200 cases); and (iv) lack of standardized metric reporting formats. These deficiencies were most prevalent in high- and unclear-risk studies, consistent with prior evidence from systematic reviews of machine learning in healthcare (Sounderajah et al., 2021; Liu et al., 2019).

Finally, this section would benefit from including confidence intervals and formal effect size estimates to contextualize differences across bias strata. Without these, claims about “superior” or “more stable” performance remain largely descriptive and potentially misleading.

## 4 Discussion

The analyzed studies demonstrate generally high diagnostic performance of AI models for classifying ovarian masses using B-mode TVS. Most studies achieved AUC values ranging from 0.85 to 0.95, with sensitivity and specificity typically above 80% (Acharya et al., 2018; Sadeghi et al., 2024). However, substantial heterogeneity across studies suggests caution when interpreting pooled performance metrics due to sample size, class balance, and data quality variations.

Automatic segmentation demonstrated statistically significant accuracy and sensitivity superior to manual segmentation, likely due to reduced observer variability (*p* = 0.007 and *p* = 0.042, respectively). Although manual segmentation provides potentially optimal delineation by experts, it is prone to operator-dependent biases. Automatic segmentation offers reproducibility and scalability but introduces errors if segmentation quality is suboptimal (Chiappa et al., 2021). Given these findings, future research should explicitly compare different segmentation algorithms and validate them against expert delineation standards to enhance clinical applicability.

Contrary to expectations, dataset size alone showed no clear correlation with diagnostic performance within datasets ≤5,000 images. Small datasets sometimes reported exceptional accuracy, likely due to extensive data augmentation strategies, while larger datasets faced increased variability and complexity, offsetting potential accuracy gains (Acharya et al., 2018; Liu et al., 2019). Thus, methodological rigor, dataset diversity, and validation strategy appear more influential than dataset volume alone.

Comparison among model architectures (classical ML, CNNs, and Transformers) revealed no consistent superiority. Traditional ML models and simple neural networks occasionally matched the performance of sophisticated CNNs and Transformers (Acharya et al., 2018). Although CNN-based models showed visually higher median accuracy and reduced variance, no statistically significant differences were observed across architectures (*p* = 0.258). Superior results often correlated with modern methodological standards rather than architectural innovation alone. Future research should directly compare these architectures under standardized experimental conditions to clarify intrinsic performance differences clearly.

The risk of bias significantly affected the result’s validity. Studies with high or unclear bias frequently reported inflated performance metrics with notable dispersion (e.g., specificity SD = 11.7%) due to methodological weaknesses like internal-only validation, small sample sizes, and lack of external validation (Sounderajah et al., 2021; Roberts et al., 2021). Such biases undermine clinical generalizability, emphasizing the need for rigorous validation standards (e.g., TRIPOD-AI, STARD-AI) and multicenter, prospective validation.

Temporal analysis showed methodological evolution influencing perceived performance gains. Despite less sophisticated techniques, earlier studies occasionally reported superior outcomes due to less rigorous validation methods, whereas recent studies employed stricter evaluation, tempering observed performance improvements (Sounderajah et al., 2021; Roberts et al., 2021). Therefore, performance comparisons across time should account for these evolving methodological contexts.

Recent advances emphasize the critical importance of precise methodological design and targeted biological understanding. For instance, Liu et al. (2024) highlighted how epitope-specific targeting of HER2 significantly influences therapeutic outcomes in solid tumors, illustrating the necessity of methodological precision in clinical efficacy. Similarly, Ma et al. (2023) underscored the potential benefits of integrating detailed molecular insights into advanced diagnostics by showing how the glycolytic enzyme ENO1 modulates choline phospholipid metabolism and tumor proliferation. Such multidisciplinary integration may substantially improve cancer characterization and patient outcomes.

AI currently demonstrates potential as an adjunctive diagnostic tool rather than a standalone solution. Real-world applicability requires rigorous external validations, standardized metric reporting (e.g., sensitivity, specificity, predictive values), and integration within clinical workflows. Moreover, evaluating the clinical impact of AI, particularly for distinguishing borderline ovarian lesions, remains essential due to their clinical complexity and diagnostic challenges (Roberts et al., 2021).

Given the limited reporting of comprehensive metrics like AUC and F1-score, future studies should ensure the consistent inclusion of these metrics to improve clinical interpretability and decision-making.

In conclusion, while AI models for ovarian mass classification via TVS demonstrate promising diagnostic accuracy, significant methodological limitations currently restrict their clinical translation. Future research must prioritize external validation, robust methodological standards, multidisciplinary integration, and transparent, standardized reporting. Prospective, multicenter studies remain crucial to fully validate these models’ clinical utility, generalizability, and real-world applicability.

## 5 Conclusion

AI models applied to B-mode TVS images demonstrate strong diagnostic performance for classifying ovarian masses, achieving high sensitivity, specificity, and overall discriminative ability (AUC). Automated segmentation significantly outperformed manual methods in accuracy and sensitivity, likely due to enhanced standardization and reduced inter-observer variability.

Nevertheless, these findings must be interpreted cautiously due to considerable methodological heterogeneity, variations in dataset size and quality, and significant risks of bias identified among several studies. Additionally, no consistent superiority emerged among different AI architectures (CNNs, classical ML, or Transformers), suggesting that methodological rigor, validation procedures, and data standardization may be more influential determinants of performance than the specific model architecture itself.

Future research should prioritize prospective, multicenter external validation under realistic clinical conditions for reliable clinical translation. Moreover, rigorous adherence to standardized reporting guidelines (e.g., TRIPOD-AI, STARD-AI), comprehensive metric reporting, including sensitivity, specificity, AUC, and F1-score, and explicit evaluation of clinical utility, especially in distinguishing borderline ovarian lesions, are essential.

In summary, while AI holds significant promise for OC diagnosis using TVS, overcoming current methodological limitations through robust validation and standardized methodological practices is imperative for successful integration into clinical practice.

## Data Availability

The original contributions presented in the study are included in the article/Supplementary material, further inquiries can be directed to the corresponding author.
